# Enhanced metagenomic surveillance for bovine respiratory disease pathogens and antimicrobial resistance by hybridization capture sequencing

**DOI:** 10.1128/aem.00977-25

**Published:** 2025-09-10

**Authors:** Jennifer N. Russell, Daniël Kos, Elhem Yacoub, Ashton N. Sies, Brian Warr, Murray Jelinski, Antonio Ruzzini, Andrew D. S. Cameron

**Affiliations:** 1 Department of Biology, University of Regina 98642, Regina, Saskatchewan, Canada; 2 Institute for Microbial Systems and Society, Faculty of Science, University of Regina 98641, Regina, Saskatchewan, Canada; 3 Department of Large Animal Clinical Sciences, Western College of Veterinary Medicine, University of Saskatchewan 70399, Saskatoon, Saskatchewan, Canada; 4 Department of Veterinary Microbiology, Western College of Veterinary Medicine, University of Saskatchewan 70399, Saskatoon, Saskatchewan, Canada; 5 Department of Biochemistry, Microbiology and Immunology, College of Medicine, University of Saskatchewan 12371, Saskatoon, Saskatchewan, Canada; Washington University in St. Louis, St. Louis, Missouri, USA

**Keywords:** bovine respiratory disease, hybridization capture sequencing, targeted metagenomics, antimicrobial resistance genes, co-infection

## Abstract

Bovine respiratory disease (BRD) is the primary disease of cattle and is responsible for most of the antibiotic use in the beef industry, both for metaphylaxis and treatment. Infection prevention and targeted treatments would benefit from detecting and identifying bacterial pathogens and, ideally, assessing antibiotic sensitivity. Here, we report success refining targeted metagenomics by hybridization capture sequencing (CapSeq) to detect and genotype bacterial pathogens and genes for antibiotic resistance in BRD. We developed a novel multi-locus sequence typing (MLST) strategy to both enhance assay sensitivity and provide epidemiological data compatible with traditional typing assays. Phylogenetically informed panel design by ProbeTools coupled with hybridization capture enabled the sequencing of novel genotypes and elucidated coinfection by distinct strains of *Mycoplasmopsis bovis*. CapSeq was orders of magnitude more sensitive than classical metagenomics at a scale compatible with low-throughput DNA sequencing capacity in diagnostic laboratories. Iterative design and testing of longer typing loci confirmed improvements in locus detection through a strategy applicable to future target selection. CapSeq detected bacterial pathogens that evaded detection by traditional metagenomics, and it demonstrated greater range and sensitivity for antibiotic resistance gene detection.

**IMPORTANCE:**

Shotgun metagenomic sequencing for infectious disease diagnostics and surveillance benefits from simultaneous detection of multiple pathogens in a sample. Adding a hybridization probe capture step to the metagenomics workflow enriches targeted loci to improve the sensitivity of pathogen detection without compromising the ability to detect pathogen variants. Our custom probe panel enables multi-locus sequence typing of bovine respiratory disease agents as well as capture of antibiotic resistance genes, which improves the sensitivity of metagenomic testing and provides genotyping data compatible with traditional assays. This study demonstrates the utility of new design principles for probe panels; it also demonstrates how targeted metagenomics provides important new insights into co-infections and is equally useful for surveillance of environmental reservoirs of disease agents.

## INTRODUCTION

Bovine respiratory disease (BRD) is the leading cause of cattle morbidity and mortality in North America (1). Despite the introduction of new vaccines and antimicrobials, rates of BRD have remained relatively stable for decades. This is partly due to the difficulty and cost of detecting and characterizing the infectious agents responsible, which prevents treatment tailored to the species present and their antimicrobial resistance capacity. BRD is a complex disease that may be initiated by viral pathogens, which allow opportunistic bacterial pathogens, primarily the mollicute *Mycoplasmopsis bovis* and three members of the *Pasteurellaceae* family, *Mannheimia haemolytica*, *Pasteurella multocida,* and *Histophilus somni* (2) to become pathogens. *M. bovis* and *H. somni* can advance to systemic infections, including septic arthritis (SA) and cardiac disease (3–5). SA further impacts animal welfare and reduces the economic output as SA is difficult to treat and accounts for half of mortalities of lame cattle (6).

BRD is associated with the stresses of animal sales and travel with the onset of disease occurring early after arrival at a feedlot. Thus, metaphylactic antimicrobial treatment of high-risk cattle upon entry to the feedlot is used to control the disease. In North America, the most common drug classes used for metaphylaxis and treatment are cephalosporins, fluoroquinolones, macrolides, phenicols, and tetracyclines (7). The emergence and prevalence of resistant organisms is an ongoing challenge to animal health and economic sustainability of the industry. In *M. bovis*, resistance is associated with modification of antimicrobial binding sites through nucleotide polymorphisms that lead to reduced sensitivity to fluoroquinolones, phenicols, tetracyclines, and macrolides (8, 9). The large number of genetic adaptations seen in field isolates of *M. bovis* associated with BRD is remarkable and is distinct from the many classes of antibiotic resistance genes (ARGs) that have evolved separately from targets to confer resistance to antibiotics. These accessory ARGs are ubiquitous in feedlot environments and are often detected in clinical BRD isolates that belong to the family *Pasteurellaceae* (3, 10–12).

Proactive culture-independent metagenomic antimicrobial resistance (AMR) surveillance approaches to detect relevant ARGs have been proposed to improve disease outcomes by facilitating informed and effective antimicrobial use. This is rationalized by the routine use of culture-based diagnostics that are both costly and time-consuming to provide real-time data for controlling ongoing outbreaks (2). Pilot studies toward scalable and practical surveillance of ARG and BRD pathogens have been completed within cattle and their pen environment (10–14). Water bowls, for instance, are communal sources of water in feedlot pens, providing focal points of cattle contact for passive surveillance of BRD pathogens and relevant ARGs (10). Nevertheless, these studies have run into several common limitations of currently established techniques for surveillance. Namely, the untargeted nature of shotgun metagenomics limits sample types due to low bacterial DNA yields. Untargeted shotgun sequencing additionally expends resources by sequencing DNA that is not of interest (host, plant genomes in the feed, etc.). This contrasts with PCR-based approaches, which are targeted and rarely advance all the way to DNA sequence information.

Hybridization probe capture sequencing, referred to here as “CapSeq,” involves enrichment of target DNA before next-generation sequencing. The length of CapSeq probes (typically 80–120 nucleotides) makes DNA:DNA hybridization tolerant to nucleotide mismatches, providing low-bias sampling of target diversity and detection of novel variants. Custom hybridization probe panels are designed to target specific genes of interest at a scale well beyond PCR multiplexing. This allows for the downstream sequencing of thousands of targets at a time, allowing for the aforementioned balance of sequence specificity and diversity to be studied without the need for ultradeep sequencing experiments. Commercially available panels are available; however, this technology can be tailored to specific applications such as BRD pathogen surveillance. In fact, capture probe design using ProbeTools software, for example, outperforms other approaches because it specifically considers diversity in pathogen clades (15). Users have ultimate control over target selection and data interpretation. This contrasts with the more common approach of tiling probes across a representative genome, followed by reliance on hybridization to capture related pathogens in a species or genus group.

Here, we present a custom panel design that targets bacterial multi-locus sequence typing (MLST) regions in addition to virulence and resistance determinants in a format to enhance detection and genotyping of BRD pathogens in complex samples and samples with low biomass. We compared the targeted CapSeq to untargeted shotgun metagenomics on water and biofilms from water bowls in conventional North American beef cattle feedlots. Additionally, a set of clinical samples from SA cases was analyzed using the CapSeq probe set. Enrichment with the BRD bacterial probe panel outperformed traditional untargeted metagenomics for detecting and genotyping pathogens, virulence genes, and ARGs in surveillance samples and performed equally well in clinical samples.

## MATERIALS AND METHODS

### Sample collections

Water and sediment samples were collected from water bowls at conventional feedlots in Alberta, Canada, in 2021. The collection of joint fluid from animals diagnosed with septic arthritis was based on clinical diagnoses by veterinarians (Animal Care Protocol #2021-0081). The live animal was sampled by arthrocentesis. Sample collection from dead animals was performed during necropsies in the field. To minimize contamination, a washed knife was used to reflect the skin from over the stifle. Contact between the subcutaneous tissue and the knife was minimized by pulling the skin with manual traction.

### BRD bacterial probe panel design and synthesis

A custom reference database of target sequences was assembled, as described in Supplemental Materials. Capture probes were designed from the reference database by the ProbeTools software (v0.1.6) (16); probe distributions across targets are presented in Table S1. Probes were synthesized at the 20,000 probe scale by Twist BioSciences (San Francisco, CA, USA).

### Nucleic acid extractions

Validation of custom BRD CapSeq probe panels was conducted with cultured isolates representing each of the four species: *M. bovis* PG45 ATCC 25523, *M. haemolytica* ATCC 33396, and *P. multocida* ATCC 12947. For *H. somni,* a clinical isolate was utilized (CCR1, NCBI GCA_049545085.1). Swabs were used to collect very small amounts of biomass for DNA extraction to mimic clinical and environmental sample volumes.

Pure bacterial cultures were grown overnight in Brain–Heart Infusion broth, then cultures were diluted 1:10 in 1.0 mL of phosphate-buffered saline, and pelleted by centrifugation for 1.5 mins at 12,000 rpm. Pellets were washed in 1.0 mL PBS media. A nasopharyngeal swab was mixed in the washed culture, and the swab tip was placed in 1.0 mL of fresh PBS. This solution was vortexed for ~10 seconds to release the bacterial cells, and 10^−1^ and 10^−2^ dilutions were made in 1.0 mL of PBS. Total nucleic acids were extracted from each dilution in a Kingfisher Duo Prime Purification System (Thermo Scientific) using the Biosprint 90 One-For-All Vet Kit (Indical Bioscience) using the Whole Blood protocol. DNA concentration was measured using a QuBit dsDNA High-Sensitivity Kit (ThermoFisher Scientific). To confirm that the extractions contained detectable bacterial DNA, 16S V4 abundance was measured by qPCR using the Perfecta SYBR Green Fast Mix with Rox (New England Biolabs).

DNA was extracted from bacterial communities in water bowl water and sediment samples using the PowerSoil DNA extraction kit (Qiagen). To process water samples, large particulates were removed by gravity flow through sterile coffee filters. The filtrate was then subjected to a second filtration step using a 0.2 μm filter (Nalgene Rapid-Flow Sterile Single Use Bottle Top Filter (595-4520)). The retentate of this filter was then recovered by removing the filter paper, vortexing it in phosphate-buffered saline, and harvesting the no longer adherent material by centrifugation. The processing of sediment samples was performed to mimic that the release of bacteria captured from water on a 0.2 μm filter. In a 50 mL volume, sediment samples were mixed thoroughly, allowed to settle for 60 minutes, and the top layer, which was devoid of large particulate, was concentrated by centrifugation prior to DNA extraction.

Synovial fluid samples were first cleared with quick centrifugation to remove fibrous or clumpy materials. Fibrin was common in the samples, and centrifugation would form a hard pellet that at times needed to be minced with sterile surgical scissors. DNA was extracted from the joint using a phenol: chloroform: isoamyl alcohol-based approach adapted from Beukers et al. (17) and Klima et al. (18). Briefly, samples and the subsequent aqueous phase of the extractions were mixed with an equal volume of phenol:chloroform:isoamyl alcohol three times before precipitation of DNA using an equal volume of isopropanol and washing with 70% ethanol (vol/vol). We find phenol-chloroform and alcohol precipitation to reliably yield the highest nucleic acid yields, which is important for the study of low biomass in synovial fluid.

### DNA library synthesis and hybridization probe capture

DNA extractions were quantified using the Qubit dsDNA HS Kit (Invitrogen); 50 ng of DNA was used for library preparation using the Twist Library Prep Enzymatic Fragmentation Kit 2.0 (Twist; 104207) and Twist Universal Adapter System – TruSeq Compatible (101308) UDI primers according to the manufacturer’s instructions. Following library preparation, libraries were quantified, and ~180 ng of each library, in sets of eight, was pooled for library hybridization. Libraries were prepared for hybridization using our custom probe panel (synthesized by Twist Biosciences, San Francisco, CA, USA) and the Twist Universal Blockers (100578) according to the manufacturer’s instructions. The pre-hybridization solution was evaporated in a Speed Vac, and hybridization was performed using the Fast Hybridization Mix and Hybridization Enhancer from the Twist Fast Hybridization and Wash Kit (104180) with the following thermal cycling conditions: 95°C for 5 minutes and 60°C for 30 minutes. Hybridized targets were then bound to streptavidin beads using the Twist Fast Wash Buffers (104180) and Twist Binding and Purification Beads (100983) according to the manufacturer’s instructions. Post-capture PCR amplification was performed using the Amplification Primers and Equinox Library Amp Mix from the Twist Fast Hybridization Kit using the following thermal cycling conditions: 98°C for 45 seconds, nine cycles of 98°C for 15 seconds, 60°C for 30 seconds, and 72°C for 30 seconds, followed by a final extension of 72°C for 1 minute and hold at 4°C; hybridized libraries were cleaned as instructed by the manufacturer, and cleaned libraries were quantified using the NEB Library Quant Kit for Illumina (NEB). To increase library molarity for sequencing, libraries generated for water and sediment samples underwent a second round of hybridization using 12 post-capture PCR cycles instead of nine, and both pools were combined and concentrated in a speed vac.

### Illumina DNA sequencing

Capture-enriched libraries were sequenced on Illumina platforms using 2 × 150-bp paired-end chemistry. Captured libraries from pure culture, water, and sediment samples were sequenced on an Illumina MiniSeq; septic arthritis samples and unenriched water and sediment samples were sequenced on an Illumina NovaSeq 6000.

### DNA read mapping

The programs FastQC (v0.11.9) (19) and MultiQC (v1.12) (20) were used to assess sequencing read quality. Adapter sequences and low-quality reads were removed by trimgalore (v0.6.10) (21) using a length of 80 and a Phred score of 30. Taxonomic identifications by Kraken2 (v2.1.2) used the standard-16 database (downloaded on 26 April 2024) or a custom taxonomic database containing reference sequences for the four bacterial BRD pathogens (22). Bowtie2 (v2.4.5) (23) or KMA (v1.4.9) (24) was used to map reads to reference sequences and the custom database used to generate the probe panels. Read alignments were visualized using Samtools (v1.15.1) (25) and Picard (v2.18.29) (26).

## RESULTS AND DISCUSSION

### A custom bacterial BRD hybridization probe panel and analysis workflow

**Fig 1:**
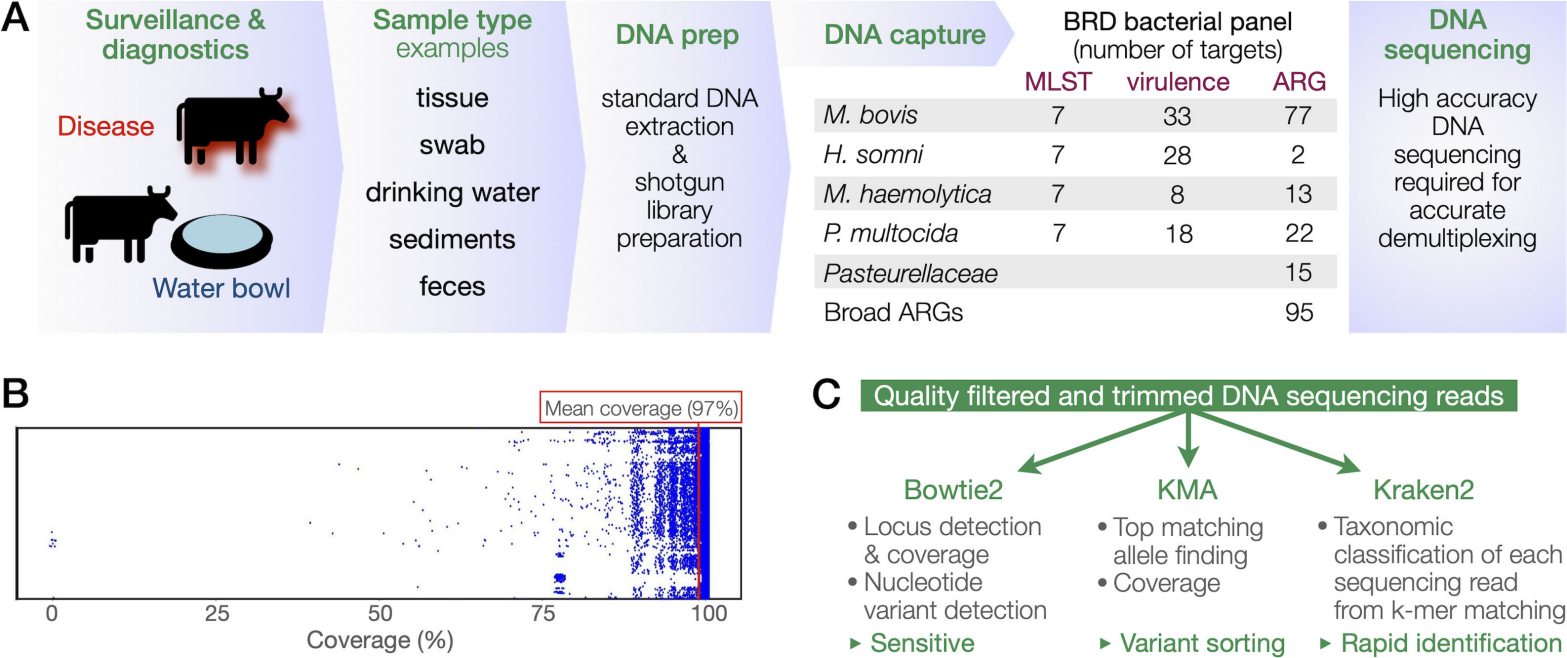
BRD bacterial pathogen custom probe. (**A**) Surveillance and diagnostic workflow for targeted metagenomic detection and genotyping of bacterial BRD pathogens and antibiotic resistance genes. CapSeq is compatible with a wide range of sample types. The present study presents data from pure culture, water, sediment, and infected tissue sample types. The table shows the number of loci targeted in each species and category. (**B**) *In silico* test of the panel’s coverage of all targets in GenBank. (**C**) DNA sequencing read analysis strategy for detection and genotyping of genes and pathogens. The functions and benefits of each analysis tool are noted.

The CapSeq workflow is illustrated in Fig. 1A. Our custom CapSeq probe panel for BRD bacterial pathogens and ARGs incorporated several important design principles that are fully described in Supplemental Materials. Importantly, probe allocation was weighted in favor of regions of higher diversity within a phylogroup, and the stoichiometries of probes were titrated to provide a greater representation of genomic regions used for detecting and genotyping pathogens. The capture.py script calculated an average of 97% coverage of variants of target loci by the custom panel (Fig. 1B), confirming that probe design effectively integrated known biological diversity and provided confidence that hybridization would capture known and unknown biological diversity.

The analysis of DNA sequencing reads from hybridization capture was conducted by parallel read-mapping strategies Bowtie 2, k-mer alignment (KMA), and Kraken2 (Fig. 1C). Locus detection and coverage was assessed by mapping DNA sequencing reads using Bowtie 2. Although Bowtie 2 alignments could be used to detect genetic variants, it is an inefficient approach for variant-calling. KMA sorts multi-mapping reads to find the best aligned reference, making KMA particularly useful for typing locus variants (24). Kraken2 (22) provided rapid taxonomic classification using pre-established or custom reference databases.

**Fig 2:**
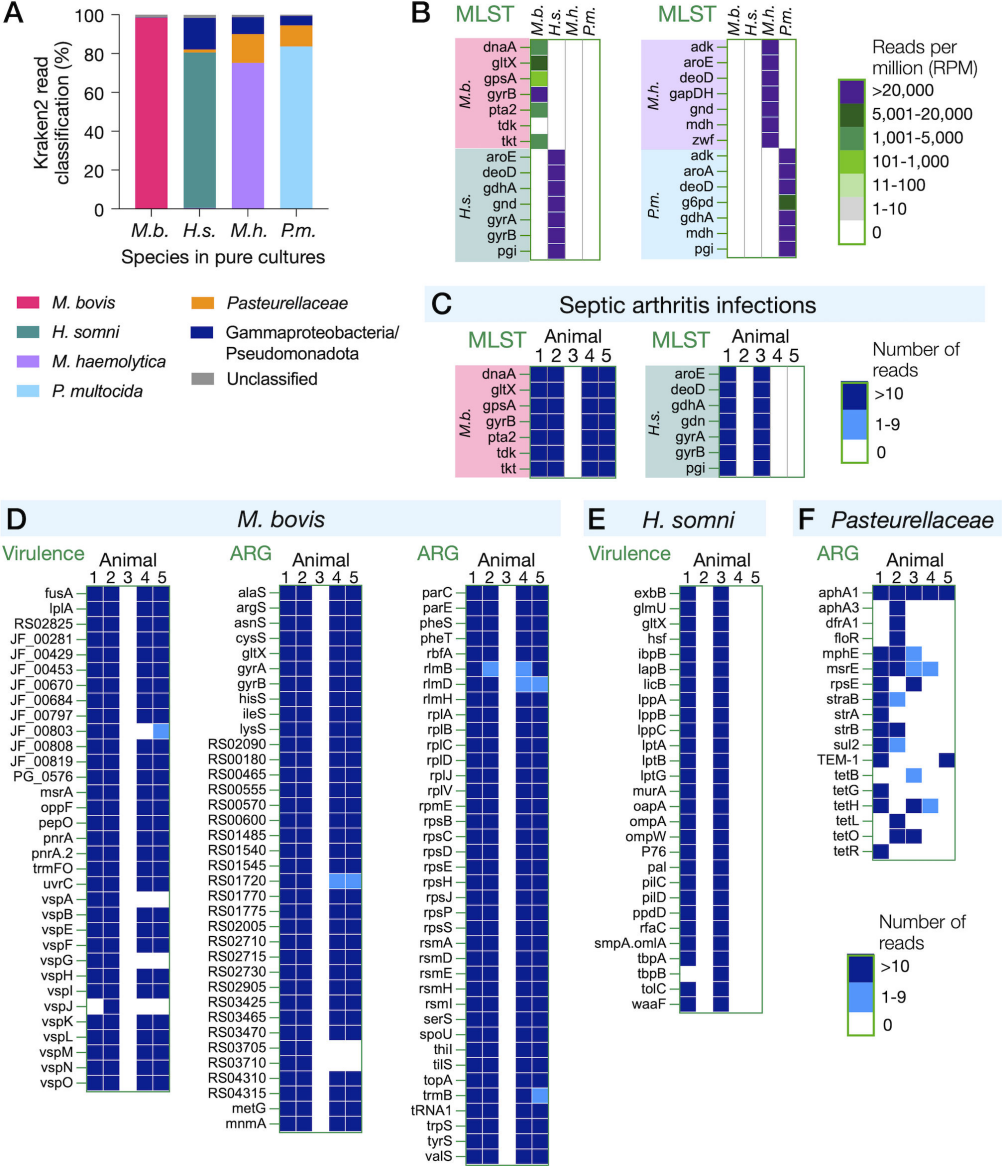
CapSeq detection and genotyping of bacterial pathogens and ARGs in synovial fluid samples from cases of septic arthritis. (**A**) Kraken2 classification of all DNA sequencing reads in CapSeq from pure cultures. *M. bovis* (*M.b*.), *H. somni* (*H.s*.), *M. haemolytica* (*M.h*.), and *P. multocida* (*P.m*.). (**B**) Read mapping to MLST loci, colored according to normalized reads per million (RPM). (**C–F**) CapSeq detection of bacterial infections in septic arthritis. Synovial fluids from septic arthritis were collected postmortem in animals #1–4 and from a living animal #5. No DNA sequencing reads mapped to *M.h*. or *P.m*. (not shown).

CapSeq using the custom BRD bacterial panel was tested first with pure cultures of each bacterial species. Rapid taxonomic classification with Kraken2 was highly specific for *M. bovis* (Fig. 2A). Kraken2 succeeded at species-level identification of over 75% of sequencing reads for three species of *Pasteurellaceae*, and the other 1% to 15% of reads were nonspecifically classified to the correct family *Pasteurellaceae* and class Gammaproteobacteria (Fig. 2A). The high percentages of DNA sequencing read classification to source species confirmed that Kraken2 is useful for rapid identification of critical BRD pathogens in metagenomic analysis.

### Multilocus sequence typing by CapSeq

MLST schemes are well-established for three of the four BRD bacterial pathogens: *M. bovis*, *M. haemolytica*, and *P. multocida*. We developed a comparable MLST scheme for *H. somni* by selecting orthologs of core genes that are used for MLST in other bacterial species, as described in Supplemental text (Fig. S1). CapSeq from pure cultures showed discrete mapping to each source species by Bowtie2 and KMA (Fig. 2B), demonstrating that the CapSeq workflow readily distinguishes between BRD pathogens. The fraction of total reads mapping to each locus was uniformly high within each species of *Pasteurellaceae*, and 100% coverage of loci enabled specific strain typing (Table S2). Conversely, *M. bovis* had a lower overall fraction of total reads mapped to the MLST loci because of the relatively larger number of additional *M. bovis* loci (~4 x) compared to the *Pasteurellaceae* in the custom probe design (Fig. 1A). Incomplete coverage of two loci, *gpsA* (98% coverage) and *tkt* (89% coverage), and no coverage of *tdk* initially prevented typing (Fig. 2B; Table S3). Additional analysis of locus mapping is provided in the Supplemental materials. We address the failed capture of the *M. bovis tdk* locus below.

### CapSeq diagnosis of joint tissue infection resolves co-infecting variants of *M. bovis*

**Fig 3:**
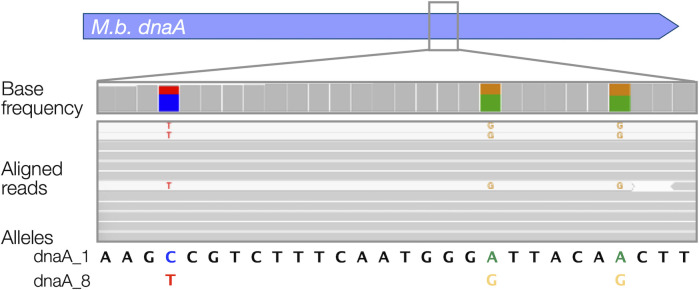
Co-infection by two *M. bovis* variants detected by CapSeq. Bowtie alignment of DNA sequencing reads in a select region of *dnaA* shows high-confidence identification of two alleles at the same locus. The top track illustrates the relative abundance of alternate bases at the three variant positions in approximately 600-fold read depth. In the DNA sequencing read alignments, reads that map to the consensus (majority) sequence are gray, whereas reads that have mismatches to the consensus are white. Colored bases in the white reads show the minority sequence. Linkage of the three variants confirms the annotation of two discrete alleles (dnaA_1 and dnaA_8) by KMA. Only a portion of *dnaA* is zoomed for simplicity.

To explore the potential utility of the CapSep panel, five samples collected from cattle that were either experiencing or that had succumbed to disease were analyzed. We rationalized that joints associated with septic arthritis (SA) would serve as a good test case because this disease is thought to be limited to few pathogens, including *M. bovis* for which the panel contained 110 targets, which could also inform on mutations related to antimicrobial use. Importantly, SA is the leading cause of lameness in North American feedlots and has a mortality rate approaching 50% (6), and culture-based diagnostics implicate *M. bovis* and *H. somni* as the leading cause. CapSeq from four postmortems (#1–4) and a living sick animal (#5) detected *M. bovis* in animals #1, 2, 4, and 5, but not animal #3 (Fig. 2C), which was confirmed by PCR. *H. somni* was detected co-infecting animal #1 and as the sole BRD pathogen in animal #3. As observed above with pure cultures, capture and read mapping were specific to *M. bovis* virulence and ARG loci (Fig. 2D) and *H. somni* virulence loci (Fig. 2E). Even though the animal #1 sample was sequenced to a great depth of over 35 million reads, no CapSeq reads mapped to *M. haemolytica* and *P. multocida* loci, providing strong evidence for the absence of these two species. Animal #2 was co-infected with two distinct sequence types of *M. bovis* as CapSeq reads mapped to distinct alleles of *M. bovis dnaA* and *tkt* (Fig. 3; Table S4). This is yet another example of the sensitivity of the CapSeq approach, allowing for discrimination between variant alleles attributed to *M. bovis*-associated disease from complex clinical materials. We note that CapSeq is capable of multistrain detection for all pathogens targeted in the panel. However, the presence of the other pathogens was relatively minor compared to *M. bovis,* and no example of multistrain infection was found for the other pathogens.

In addition to pathogen detection, a limited number of ARGs associated with *Pasteurellaceae* were detected in the SA samples (Fig. 2E). These ARGs are considered to be part of accessory genomes; however, their existence is not exclusive to the *Pasteurellaceae*. To this end, a relatively high number of gamma-proteobacterial ARGs were detected in animal #2 despite the absence of detectable *H. somni*, *M. haemolytica*, and *P. multocida* by CapSeq or PCR. Additional surveillance of live animals and cattle joint microbial community structures before and after culling is required to identify the bacterial hosts of ARGs detected in this pilot study.

The probe panel targeted 110 core *M. bovis* genes and almost 30 core *H. somni* genes in addition to MLST loci, which allowed us to compare bacterial strains based on the gene content. The *M. bovis* in postmortem samples from animals #1 and #2 were very similar in gene content, with only virulence gene *vspJ* missing from *M. bovis* in animal #1 (Fig. 2D), a pattern noted previously in *M. bovis* (27). Similarly, the *M. bovis* in postmortem sampling of animal #4 matched the gene content of *M. bovis* detected in the sick animal #5. Five *M. bovis* loci were undetected in animals #4 and #5, suggesting that five genes are accessory virulence and resistance determinants.

### CapSeq outperformed untargeted shotgun metagenomics for pathogen surveillance

**Fig 4:**
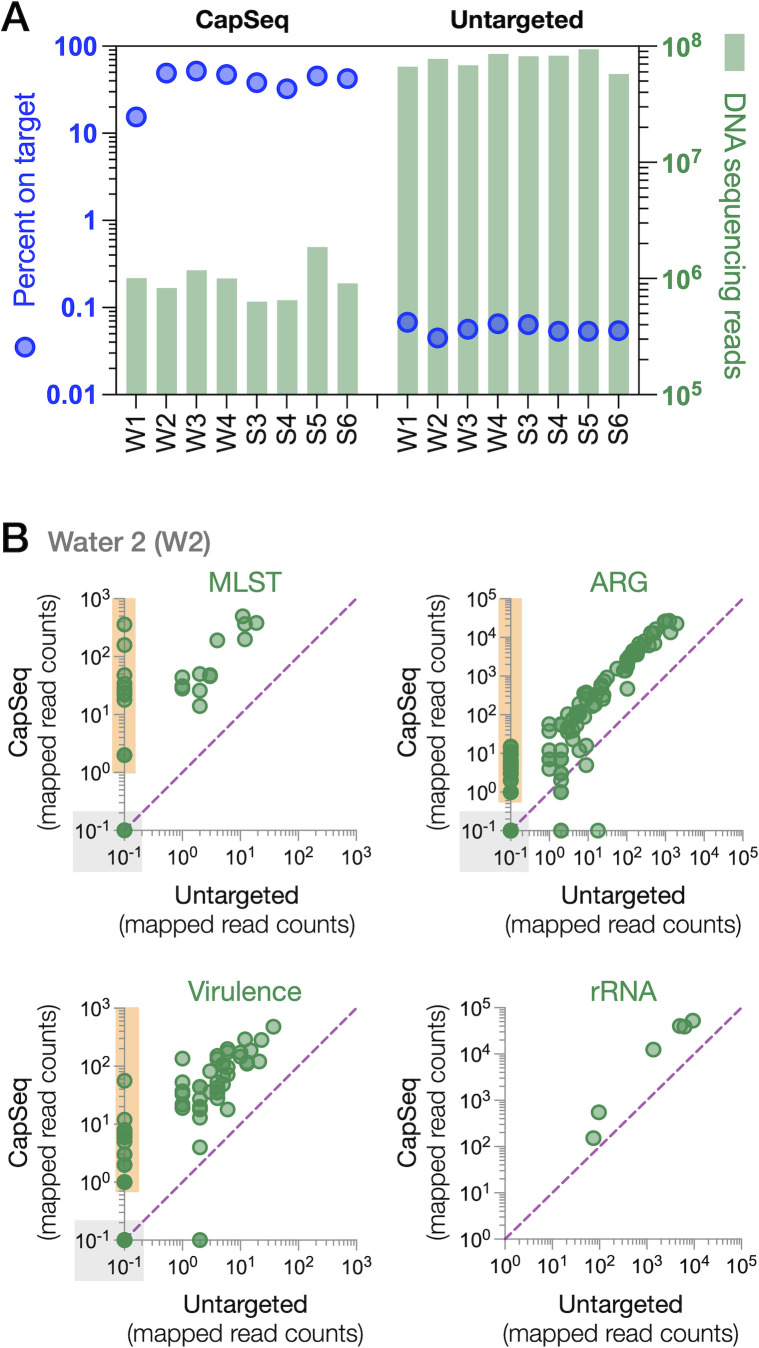
CapSeq for surveillance in environmental samples. (**A**) Percent of reads mapping to target loci (blue) and DNA sequencing depth are plotted on independent log scales. Sample numbers are differentiated by the abbreviations W (water) and S (sediment). (**B**) Number of reads mapping to target loci in CapSeq (y-axis) compared to shotgun metagenomic (x-axis). Each green circle is a locus. A dashed purple line demarcates equal reads in both techniques. Orange fill highlights loci detected by CapSeq but not detected by untargeted shotgun sequencing. Loci having 0 mapped reads are plotted on the log scales at 10^−1^, highlighted by gray fill.

Successful application of the panel to cultivated and clinical samples supported our effort to utilize the approach for surveillance from accessible but low biomass materials such as water from cattle feedlot watering bowls. Using metagenomic data from feedlot samples from water and sediment from water bowls (NCBI BioProject PRJNA978540), we tested the efficacy of CapSeq for surveillance of BRD pathogens and ARGs in water bowls. Sequencing to an average of 1.0 × 10^6^ filtered reads per library, significantly fewer than the average of 7.7 × 10^7^ filtered reads for untargeted metagenomics libraries were used as the basis of detection and comparison between approaches. Despite its much lower read depth, all four bacterial species were consistently detected in all samples with CapSeq (Fig. 4A). Using a water sample W2 as an example, the ratio of locus detection between targeted (CapSeq) and untargeted metagenomics is further illustrated. All MLST loci in the three species of *Pasteurellaceae* were detected by CapSeq (1,400 sequencing reads mapped to 21 MLST loci); three *M. bovis* MLST loci were detected by CapSeq, but none were detected in untargeted sequencing results. CapSeq performed as designed by generating more reads mapping to MLST, ARG, virulence, and rRNA loci than did untargeted metagenomics (Fig. 4B), demonstrating a greater sensitivity for low-abundance genes despite much lower sequencing effort. In all four gene classes, a positive correlation between the number of reads mapping at each locus in CapSeq and untargeted metagenomics indicated that both methods are semi-quantitative and can discriminate between low- and high-abundance genes in complex samples (Fig. 4B).

### CapSeq surveillance of ARGs in feedlot water bowls

**Fig 5:**
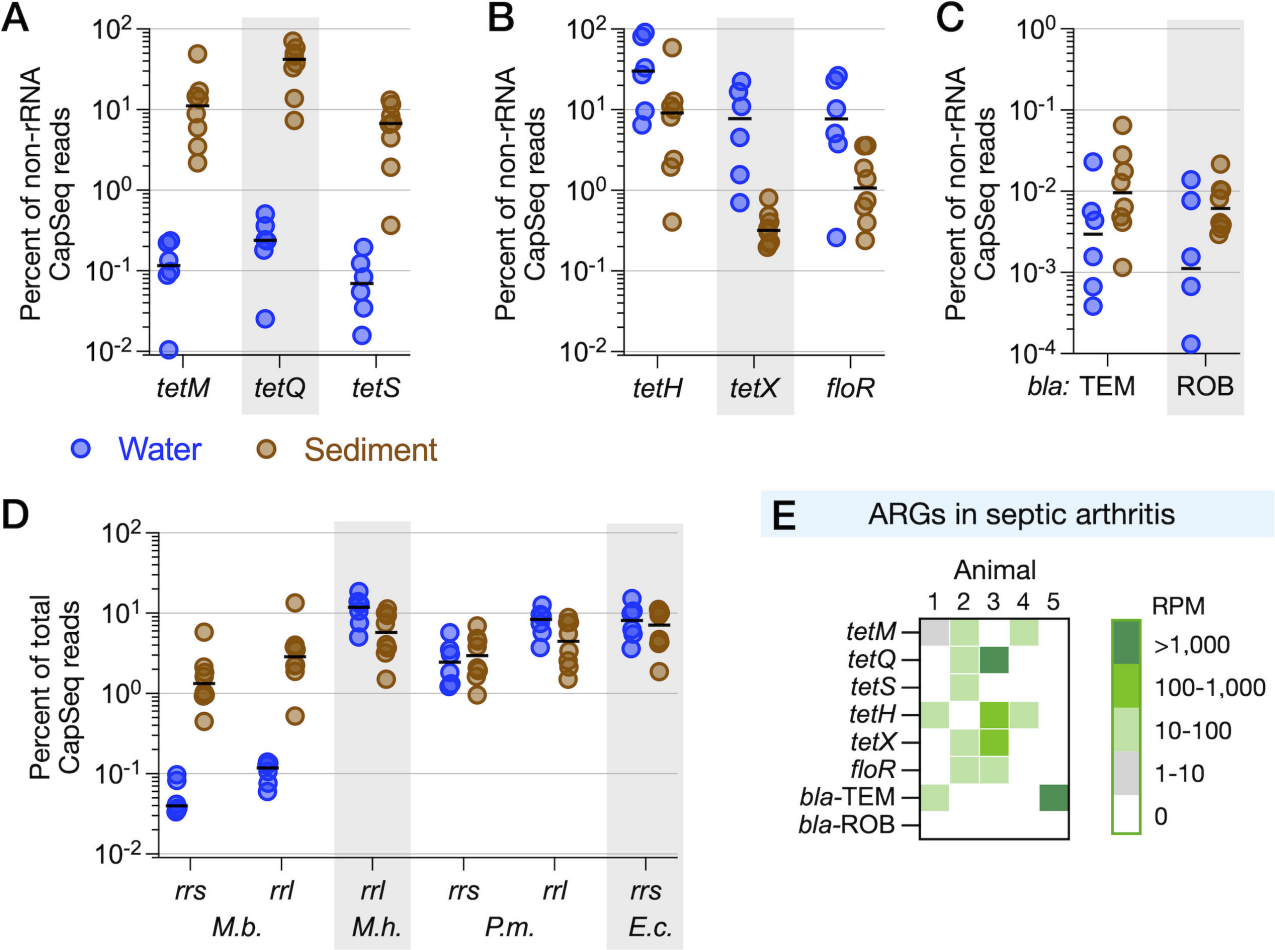
Abundances of select ARGs in water and biofilms in feedlot water bowls. (**A**) Percent of reads mapping to *tet* genes that confer ribosomal protection in water (blue) or sediment (brown). Values plotted as the percentage of non-rRNA reads. (**B**) Highly abundant resistance genes that are on average more abundant in water than sediment. (**C**) Abundance of beta-lactamase genes, which are of much lower abundance than the other genes in this figure. (**D**) Abundance of the rRNA loci represented in the CapSeq bacterial BRD probes. (**E**) Detection of the same ARGs in septic arthritis. DNA sequencing reads mapping to each locus are plotted as normalized reads per million (RPM) of non-rRNA reads; 1,000 RPM is equivalent to 0.1% of mapped reads in (**A–C**).

CapSeq confirmed significant differences in ARG distributions between water and sediment collected from water bowls in feedlot pens. Genes encoding tetracycline resistance by ribosome protection or antibiotic inactivation are prominent in agricultural and non-agricultural environments worldwide (28–31). CapSeq testing of water or sediment samples from feedlot water bowls revealed an abundance of *tet* genes, collectively accounting for an average of 75% of non-rRNA reads. There was a clear distinction between which *tet* genes dominated water or sediment samples (Fig. 5A and B). Genes *tetM, tetQ,* and *tetS* that encode ribosomal protection mechanisms in a wide range of bacterial taxa accounted for, on average, 60% on non-rRNA reads in sediment, but less than 0.5% of reads in water samples (Fig. 5A). Other tetracycline ARGs demonstrated an inverse pattern of being concentrated in water instead of sediment, including *tetH*, an efflux pump that is commonly associated with *H. somni*, and the broadly distributed *tetX*, a tetracycline-modifying enzyme (Fig. 5B). Tetracycline resistance is commonly observed in clinical *H. somni*, *P. multocida,* and *M. haemolytica* isolates, including *tet(H*) (32, 33). Resistance to macrolides encoded for by *msrE*, *mph(E), erm (42), ermF,* and *estT* within *P. multocida* and *M. haemolytica* has also been previously reported (34). Similarly, a chloramphenicol exporter gene associated with *Acinetobacter* and *Enterobacteriaceae*, *floR*, was more prevalent in water than sediment. This phenicol efflux pump is also commonly found in clinical isolates of *H. somni*, *P. multocida,* and *M. haemolytica*; however, the *in vitro* correlation of resistance and *floR* can be inconsistent (32).

Beta-lactam antibiotics are used to treat cattle and humans. Ceftiofur, a third-generation cephalosporin, is used to treat BRD at feedlots. Thus, environmental detection of beta-lactam resistance can inform modeling of connections between human and agricultural systems. Two classes of beta-lactamase genes, *bla*_TEM_ and *bla*_ROB_, were detected in water and sediment (Fig. 5C). Beta-lactam resistance gene *ROB-1* has been previously found in *H. somni* and *P. multocida* isolates. For example, ROB-1 has recently been observed on the same integrative conjugative element as *estT* in feedlot *P. multocida* isolates (35–37), and retrospective analysis of *M. haemolytica* genomes revealed a positive correlation between these two genes (38).

In the septic arthritis samples, on average, 10–100-fold fewer reads mapped to *tet* genes (Fig. 5E), in contrast to the high abundances of ARGs in water bowls. Two samples tested positive for *bla*_TEM_, but *bla*_ROB_ was not detected (Fig. 5E).

### Ribosomal RNA capture

Nucleotide substitutions in *rrs* (16S) and *rrl* (23S) ribosomal genes can confer resistance to antibiotics. Regions with known resistance mutations were included in the probe panel: *M. bovis* (*rrs*, *rrl*), *M. haemolytica* (*rrl*), *P. multocida* (*rrs*, *rrl*), and *E. coli* (*rrs*). The 16S variable region sequences are widely used to identify bacterial genera, and hybridization capture has been applied to 16S targets (39, 40). rRNA loci present a challenge in metagenomic analyses because the loci consist of informative variable regions alternating with highly conserved regions. We anticipated that conserved regions would preclude genus-level resolution in the targeted metagenomes due to high similarity across high taxonomic ranks. We observed that CapSeq readily distinguished *M. bovis* rRNA loci from the other BRD pathogens, but rRNA loci of *Pasteurellaceae* could not be effectively discriminated by the CapSeq BRD bacterial probe set (Fig. S2A). *E. coli rrs* was included in the panel for its role in antibiotic resistance. CapSeq of *H. somni* and *M. haemolytica* pure cultures resulted in alignment of ~10% of 16S rRNA reads to *E. coli rrs*, consistent with the >88% similarities between the rRNA sequences (Fig. S2B).

In water surveillance samples, CapSeq consistently detected 20-fold greater relative abundances of *M. bovis rrs* and *rrl* in sediment than in water (Fig. 5D). This may have resulted from sample processing and the ability of *M. bovis* to cross 0.2 μm filters. In contrast to the stratified distribution of *M. bovis* rRNA loci in water bowls, CapSeq read abundances at *Pasteurellaceae* and *Escherichia* rRNA loci were strikingly consistent across water and sediment samples (Fig. 5D). Regions of the *M. bovis* 16S and 23S genes are of utmost importance for tetracycline and macrolide resistance (8, 9), and AMR-associated polymorphisms were detected in SA infections by CapSeq. *M. bovis rrs* has a high frequency of polymorphisms A956 and A958 in beef and dairy cattle operations (9, 41, 42), which was detected by CapSeq in all septic arthritis tissues and surveillance samples.

### Refining the BRD bacterial probe panel

**Fig 6:**
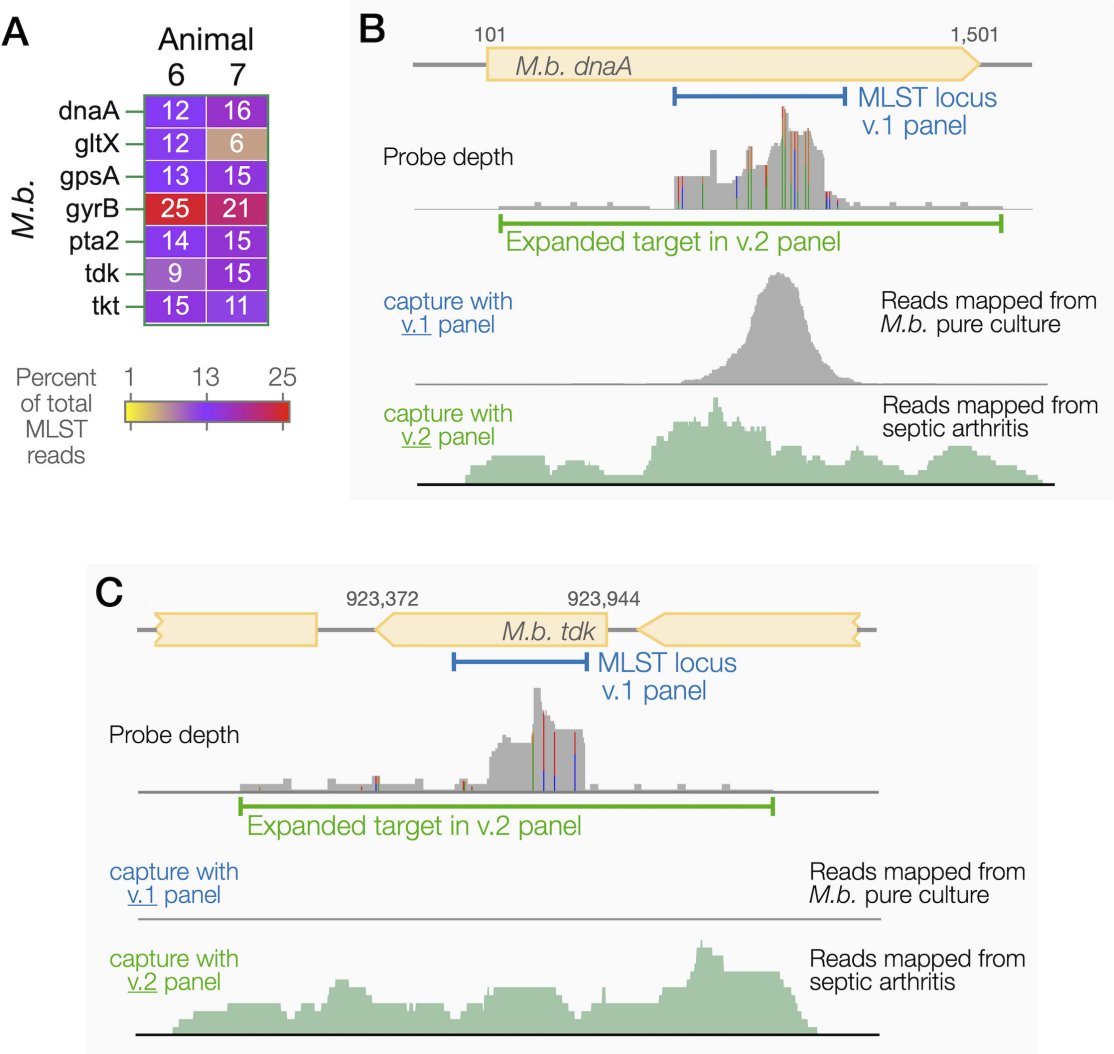
CapSeq in synovial fluid samples from cases of septic arthritis using the v.2 panel. (**A**) DNA sequencing read distribution at *M. bovis* (M.b.) MLST loci. All reads mapping to MLST were summed, and the percentage mapping to each locus is displayed as a heatmap. (**B and C**) Probe distribution and read mapping across example genes *M. bovis dnaA* and *tdk*. Gray numbers above the open reading frames indicate base positions in the genome. In the top panel showing probe depth, colored vertical strips indicate nucleotide variants at specific positions; gray vertical strips indicate the sequence identity across all known variants. DNA captured by the version v.1 panel was sequenced with 2 × 150 bp paired-end reads, whereas v.2 was sequenced with 2 × 300 bp reads.

A benefit of CapSeq is the iterative design of probe panels; for example, the number of probes and regions targeted can be expanded as required. In the first version of our custom panel, MLST targets were defined according to the established locus dimensions. However, we observed variability in capture between loci within a species, as illustrated by the absence of capture of *M. bovis tdk* in pure culture (Fig. 2B) and less than 0.8% of total MLST reads mapping to *tdk* in septic arthritis samples (Fig. S3). We produced a second version (v.2) of the panel that expanded the MLST target regions from 500 to 700 bp to at least 1.5 kb in length and then conducted CapSeq on infected synovial fluid from two live cattle suffering from SA, animals #6 and #7. CapSeq found that both animals were infected with *M. bovis*, without detectable *H. somni*, *M. haemolytica*, or *P. multocida*. With the second version of the panel, *M. bovis tdk* became detectable to a level balanced with the other MLST loci in *M. bovis* (Fig. 6A); *gyrB* consistently received a larger portion of reads (>20% of MLST reads) because two loci were targeted within this long gene, one for MLST and the other as a sequence determinant of antibiotic resistance. Comparing the read mapping between v.1 and v.2 panels demonstrated how effectively the addition of flanking probes improved the capture of the MLST loci (Fig. 6B and C). The *tdk* MLST region and targeting probes contained G + C content consistent with the genomic average, and there were no detectable sequence features that could explain the absence of *tdk* capture with the v.1 panel.

### Conclusion

Antibiotics are relied on for metaphylaxis and treatment of BRD and SA, but targeted medication is hampered by a lack of near real-time infectious disease diagnostics and surveillance. CapSeq has the potential to fill a gap in veterinary infectious disease diagnostics, bridging the gap between the limited scope of traditional culture- and PCR-based diagnostics versus data-heavy shotgun metagenomics. CapSeq has the significant advantage over culture methods and PCR, in that it can detect multiple species and ARGs simultaneously as well as a wide range of genetic variants for each target in a single clinical sample. Moreover, an average of 40% of filtered CapSeq reads mapped to the targeted loci, outperforming the sensitivity of shotgun metagenomics, in which only 0.057% of filtered reads mapped to target loci. CapSeq does not require additional techniques beyond regular DNA sequencing workflows; however, CapSeq has the added costs of probe synthesis and extra hours required for the hybridization and subsequent amplification steps.

As with untargeted metagenomics, current CapSeq approaches cannot assign widely conserved sequences, like mobile ARGs, to specific bacteria. The enrichment of targets by CapSeq precludes the general study of microbial communities: metagenome-assembled genomes (MAGs) cannot be assembled, enzymatic potential cannot be analyzed, and nontargeted pathogens are more likely to be overlooked. Although CapSeq probe panel design is limited to the current knowledge of DNA sequences, ProbeTools enables inclusion of all known sequence variants in the capture probes, and hybridization ensures capture of a wide range of genetic variants—both known and unknown—from members of target species. Enriching DNA or cDNA from target species also simplifies bioinformatic analysis. These advantages of CapSeq succeeded in sensitive detection of BRD pathogens and ARGs in diverse sample types and provided important new epidemiological insights into pathogen diversity and co-infections.

## Data Availability

DNA sequence data have been made publicly available through the National Center for Biotechnology Information: PRJNA1122138 (isolate enrichment data), PRJNA978540 (metagenomics), PRJNA1122166 (CapSeq data from water bowls), and PRJNA1140205 (CapSeq data from septic joints).

## SUPPLEMENTAL MATERIAL

File S1 aem.00977-25-s0001.pdf Supplemental methods, Tables S1 to S4, and Fig. S1 to S4.

File S2 aem.00977-25-s0002.txt Fasta format sequences of all v1 probes.

File S3 aem.00977-25-s0003.txt Fasta format sequences of all v2 probes.

ASM does not own the copyrights to Supplemental Material that may be linked to, or accessed through, an article. The authors have granted ASM a non-exclusive, world-wide license to publish the Supplemental Material files. Please contact the corresponding author directly for reuse.
