# Supplementary material for: Enhanced metagenomic surveillance for bovine respiratory disease pathogens and antimicrobial resistance by hybridization capture sequencing
Source: Appl Environ Microbiol. 2025 Sep 10;91(10):e00977-25. doi: 10.1128/aem.00977-25 (PMC12542742; doi:10.1128/aem.00977-25)
Supplement: File S1 — Supplemental methods, Tables S1 to S4, and Fig. S1 to S4. [file aem.00977-25-s0001.pdf]

Supplemental Materials

Materials and Methods – pages 2-3

Supplemental Findings – page 4

Supplemental Tables – pages 5-7

Table S1. Overview of the design of the bovine respiratory disease probe capture panel design version 1.

Table S2. Identity, coverage, gaps and PubMLST allele numbering of multilocus sequence typing targets detected from DNA extracted from the monoculture of corresponding bacteria isolated from the upper respiratory tract and lung tissue of feedlot cattle.

Table S3. Multilocus sequence typing of bacteria detected with CapSeq in the DNA extracted from monocultures of corresponding bacteria isolated from the upper respiratory tract and lung tissue of feedlot cattle. For *M. haemolytica, P. multocida*, and *M. bovis*, the established allele designations (_number) are indicated, as determined by PubMLST.

Table S4. Multilocus sequence typing of *Mycoplamopsis bovis* detected via CapSeq within the synovial joint fluid of cattle.

Supplemental Figures – pages 8-9

Figure S1. Inter- and intra-species ranges of genetic diversity at loci selected for *H. somni* MLST.

Figure S2: CapSeq detection of ribosomal genes. (**A**) Several *rrs* and *rrl* genes from the four BRC bacterial species were targeted by the custom BRD probe sets. (**B**) The calculations of coverage and percent identity between *E. coli* 16S and the four BRD bacterial pathogens.

Figure S3: CapSeq in synovial fluid samples from cases of septic arthritis using the v.1 panel. DNA sequencing read distribution at *M. bovis* (M.b.) MLST loci. All reads mapping to MLST were summed and the percentage mapping to each locus are displayed as a heatmap.

Figure S4: CapSeq detection of ARGs and virulence genes. The percentage of total CapSeq reads from pure cultures mapping to the target sequences in BRD bacterial panel v1. Read mapping to antibiotic resistance loci (**A**) and virulence genes (**B**), colored according to reads per million (RPM) mapping to each locus. Locus names are listed alphabetically in each species. Color coding of locus names is as follows: *M. bovis* (*M.b.*), red fill; *H. somni* (*H.s.*), green fill; *M. haemolytica* (*M.h.*), purple fill; *P. multocida* (*P.m.*), blue fill; and general *Pasteurellaceae*, grey fill.

Additional supplemental files

Supplemental Material 2, v1 probes: Fasta format sequences of all v1 probes

Supplemental Material 3, v2 probes: Fasta format sequences of all v2 probes

Supplemental Materials and Methods

Design principles of a custom BRD bacterial probe panel

Our custom probe panel CapSeq for BRD bacterial pathogens and ARGs incorporated several design principles. Importantly, probes were designed by the ProbeTools software (1), which improves CapSeq's key ability to detect known and unknown genetic variants by weighting probe allocations in favour of regions of higher diversity within a phylogroup. ProbeTools achieves this by aligning all sequence information provided to it, then designs probes to maximize representation of genetic variants. In other words, ProbeTools generates a phylogenetically informed panel for detecting known and unknown variants by reducing probe space allocated to conserved genomic regions.

A second strategy was implemented following ProbeTools probe selection to increase sensitivity for low abundance pathogens. Instead of equal representation of each unique probe on the panel, the stoichiometries of probes were titrated to provide greater representation of genomic regions for detecting and genotyping pathogens. Each probe targeting a bacterial typing locus was represented at a 5-fold greater concentration, ARG probes at 2-fold greater concentration, and virulence genes at standard concentration (single copy of each unique oligo) (**Table S1**). Probes targeting cattle genes and SARS-CoV-2 sequences served as positive and negative controls, respectively, and were represented at normal 1-fold concentration.

Probes were synthesized as single stranded DNA for exacting synthesis standards and for stability during long term storage. Moreover, ssDNA probes can be long (125-mer), allowing for increased hybridization stringency to capture molecules matching >75% (2). The low relative sequence complexity of a small synthesis scale (20,000 probes) can further enhance detection by providing the highest relative concentration of each unique probe sequence in the panel, reducing interference between competing probes, and ultimately reducing background capture during hybridization.

MLST strategy

MLST schemes are well-established for molecular epidemiology of veterinary and human pathogens, though BRD pathogen *H. somni* lacked an established MLST schemes, prompting us to develop a *H. somni* MLST scheme de novo. We specifically avoided tiling whole genomes from a small number of reference strains as this fails to consider real biological diversity and assigns probes to accessory genes that may be very rare in nature, even though tiling reference sequences remains the standard design strategy of commercial probe sets. Indeed, metagenomic detection and assembly of whole genomes for molecular epidemiology is impractical because of genome size and the high proportions of transient accessory genes that result in dynamic genome sizes and arrangements. Strategic design to include known genetic diversity at specific loci combined with the benefit of hybridization of long DNA molecules enabled both sensitive detection and genotyping.

MLST targets

Pasteurella multocida, Mannheimia haemolytica, and *Mycoplasmopsis bovis.* have established genes for which alleles are available at PubMLST (pubmlst.org). All defined variants were downloaded from PubMLST for the following loci: *P. multocida* (*aroA, adk, deoD, g6pd, gdhA, mdh* and *pgi*); *M. haemolytica* (*adk, aroE, deoD, gapDH, gnd, mdh,* and *zwf*); *M. bovis* (*dnaA, gltX, gspA, gyrB, pta2, tdk,* and *tkt*). In the absence of an MLST scheme for *Histophilus somni*, we selected the orthologs of loci for other members of the family *Pasteurellaceae*: *P. multocida* and *M. haemolytica*. Diversity at these loci was compared within *H. somni* and within the other members of the family *Pasteurellaceae* using NCBI BLAST (**Fig. S1**).

The Comprehensive Antibiotic Resistance Database (CARD) (23) was used to compile ARG sequences from *H. somni*, *M. haemolytica*, *P. multocida* and other *Pasteurellaceae*. Most of these ARGs are accessory genes, but a few are alleles of core genes. For the accessory/virulence and AMR components of the panel, the literature was searched for relevant accessory and AMR genes related to the target organisms. Additionally, AMR genes that confer resistance to commonly used antimicrobials within the cattle industry were also selected for the panel. Locus sequences were obtained from NCBI and CARD, and then aligned in nBLAST to obtain all genetic diversity for that gene; to ensure that only target genes were obtained, alignments of < 97 % were discarded. Finally, two positive controls and one negative control was added to the panel. For the positive controls, the *cytb* gene and a 1,200 bp region of the *gapgh* gene of *Bos taurus* were added, and the entire SARS-CoV-2 genome was added as a negative control due to fact that this virus cannot infect cattle, and because, at the time of creating the panel, SARS-CoV-2 genomes had been sequenced in the same lab space, and acted as a good indicator for contamination.

MLST is traditionally conducted by Sanger sequencing short (<700 bp) segments of seven or eight highly conserved protein coding genes in a bacterial species. Such a small number of short genomic regions with limited yet informative diversity is readily covered by a small number of CapSeq probes (**Table S1**).

All *H. somni* sequences available at GenBank, including genome contigs and PCR amplicon sequences, displayed a sequence identity distribution (>87%) within the species (green in **Fig. S1**) that did not overlap with the distribution observed for the same loci from other *Pasteurellaceae* and other bacterial species (grey in **Fig. S1**). Some *Pasteurellaceae* loci were sufficiently similar (>75% identity) to potentially be captured by hybridization; nevertheless, the distinct sequences of *H. somni* loci ensured that *H. somni* could be detected and genotyped in captured DNA sequences.

Supplemental Findings

Detection of virulence genes and ARGs through probe capture successfully matched reference genomes.

A feature of the BRD CapSeq panel is the strategic targeting of loci across multiple categories (MLST, virulence, ARG and ribosomal loci (rDNA)) to achieve phylogenetically-informed capture of biological diversity while avoiding probe dilution with less informative genomic regions. Virulence gene targets were compiled by literature searching for experimentally validated (or implicated) virulence genes in the four species. CapSeq of the four pure bacterial cultures showed these virulence genes to be well represented and detectable in reference strains: 33/33 (100%) of *M. bovis* loci, 28/28 (100%) of *H. somni* loci, 8/8 (100%) of *M. haemolytica* loci, and 13/13 (100%) of *P. multocida* loci. An additional five accessory virulence genes (*bcbD*, *dcdF*, *ecbJ*, *tadD*, *toxA*) that occur in one or only a few *P. multocida* strains were included in the panel for expanded capabilities. All virulence genes were specific to the species in which each gene was described (**Fig. S4B**); thus, their inclusion on the probe panel expanded the genomic sequence space contributing to pathogen detection and genomic epidemiology.

The ARGs listed in **Table S2** include all ARGs annotated in the four BRD bacterial species plus ARGs from any bacterial species that confer resistance to antibiotics used for prophylaxis or treatment in cattle. All 77 *M. bovis* ARGs in the panel are polymorphisms in chromosomal genes, which are anticipated to be shared by most strains of *M. bovis*. A single ARG, *rbfA*, had zero reads map from pure culture *M. bovis* capture (**Fig. S4**), but the locus was detected in the deeper reads of septic arthritis infections (**Fig. 2**). As observed with MLST, several *M. bovis* ARGs demonstrated low numbers of sequencing reads captured and mapped (**Fig. S4**), implicating inefficient hybridization capture of these specific loci.

In *M. haemolytica* and *P. multocida*, the only ARGs detected in these strains were resistance alleles of core genes *gyrA, gyrB, parC*, and *rpsE* (**Fig. S4**). Like *H. somni gyrA*, which can be treated as an MLST and an AR locus, the core genes of *M. haemolytica* and *P. multocida* in **Figure S4** can equally serve as MLST loci because they demonstrated excellent discriminating power, mapping uniquely to their cognate species. The *H. somni* test strain stood out because it was positive by CapSeq for numerous accessory ARGs annotated in *Pasteurellaceae* spp. (**Fig. S4**). ARGs that are spread and actively shared across bacterial phyla naturally do not identify the presence of any individual species when detected in surveillance.

References

1. Kuchinski KS, Duan J, Himsworth C, Hsiao W, Prystajecky NA. (2022) ProbeTools: designing hybridization probes for targeted genomic sequencing of diverse and hypervariable viral taxa. *BMC genomics* 23:579.
2. Briese T, Kapoor A, Mishra N, Jain K, Kumar A, Jabado OJ, Lipkin WI. (2015) Virome Capture Sequencing Enables Sensitive Viral Diagnosis and Comprehensive Virome Analysis. *mBio* 6:10.1128/mbio.01491-15. https://doi.org/10.1128/mbio.01491-15

Supplemental Tables

Table S1. Overview of the design of the bovine respiratory disease probe capture panel design version 1.

BRD Bacterial Panel v1			
**Category**	**Target**	**# of targets**	**# of probes**
MLST	*Histophilus somni*	7	
	Mannheimia haemolytica	7	
	Pasteurella multocida	7	
	Mycoplasmopsis bovis	7	
	**Total**	28	1,684 x 5
Virulence genes	*Histophilus somni*	28	
	Mannheimia haemolytica	8	
	Pasteurella multocida	18	
	Mycoplasmopsis bovis	33	
	**Total**	87	3,824
ARGs and resistance polymorphism sites	*Pasteurellaceae*	52	
	Mycoplasmopsis bovis	77	
	Broad AMR targets	95	
	**Total**	224	3,537 x 2
Controls	*Bos taurus cytB*	1	
	Bos taurus GAPDH	1	
	SARS-CoV-2 loci	4	
	**Total**	6	312

Table S2. Identity, coverage, gaps and PubMLST allele numbering of multilocus sequence typing targets detected from DNA extracted from the monoculture of corresponding bacteria isolated from the upper respiratory tract and lung tissue of feedlot cattle.

Organism	Locus	PubMLST allele or Genbank	Identity	Coverage	Gaps
Histophilus somni	aroE	CP042983.1	100	100	0
	deoD	CP042983.1	100	100	0
	gdhA	CP042983.1	100	100	0
	gnd	CP043001.1	100	100	0
	gryA	CP043001.1	100	100	0
	gyrB	CP043001.1	100	100	0
	pgi	CP043001.1	100	100	0
Mannheimia haemolytica	adk	2	100	100	0
	aroE	1	100	100	0
	deoD	1	100	100	0
	gapDH	1	100	100	0
	gnd	2	100	100	0
	mdh	1	100	100	0
	zwf	2	100	100	0
Pasteurella multocida	adk	16	100	100	0
	aroA	19	100	100	0
	deoD	14	100	100	0
	g6pd	26	100	100	0
	gdhA	6	100	100	0
	mdh	12	100	100	0
	pgi	6	100	100	0
Mycoplasmopsis bovis	dnaA	1	100	100	0
	gltX	2	100	100	0
	gpsA	4	97.8	97.8	0
	gyrB	2	100	100	0
	pta2	1	100	100	0
	tdk	Not detected	NA	NA	NA
	tkt	?	88.7	88.7	0

Table S3. Multilocus sequence typing of bacteria detected with CapSeq in the DNA extracted from monocultures of corresponding bacteria isolated from the upper respiratory tract and lung tissue of feedlot cattle. For *M. haemolytica, P. multocida*, and *M. bovis*, the established allele designations (_number) are indicated, as determined by PubMLST.

Organism	PubMLST	Loci
Histophilus somni	Not established	aroE, deoD, gdhA, gnd, gyrA, gyrB, pgi
Mannheimia haemolytica	ST2	adk_2, aroE_1, deoD_1, gapDH_1, gnd_2, mdh_1, zwf_2
Pasteurella multocida	Unregistered	adk_16, aroA_19, deoD_14, g6pd_26, gdhA_6, mdh_12, pgi_6
Mycoplasmopsis bovis	Unknown	dnaA_1, gltx_2, gpsA_4, gyrB_2, pta2_1, tdk_?, tkt_?

Table S4. Multilocus sequence typing of *Mycoplamopsis bovis* detected via CapSeq within the synovial joint fluid of cattle.

Allele	Identity	Coverage	Gaps
dnaA_1	100	100	0
dnaA_8	100	100	0
gltX_1	100	100	0
gpsA_3	100	100	0
gyrB_3	100	100	0
pta2_5	100	100	0
tdk_3	100	100	0
tkt_4	100	100	0
tkt_1	100	100	0

Supplemental Figures

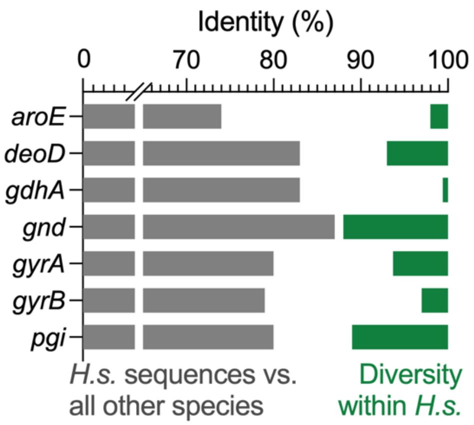

Figure S1. Inter- and intra-species ranges of genetic diversity at loci selected for *H. somni* MLST.

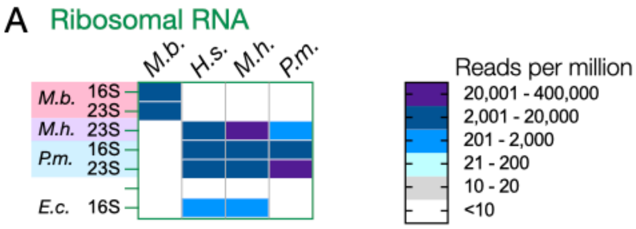

B Region of *E. coli* 16S locus included in BRD pathogen panel compared to 16S loci in BRD pathogens

Organism	coverage_%	identity_%
H. somni	100	87-89
P. multocida	99	89-90
M. haemolytica	99	88-89
M. bovis	99	77-78

Figure S2: CapSeq detection of ribosomal genes. (**A**) Several *rrs* and *rrl* genes from the four BRC bacterial species were targeted by the custom BRD probe sets. (**B**) The calculations of coverage and percent identity between *E. coli* 16S and the four BRD bacterial pathogens.

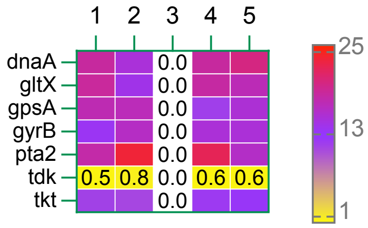

Figure S3: CapSeq in synovial fluid samples from cases of septic arthritis using the v.1 panel. DNA sequencing read distribution at *M. bovis* (M.b.) MLST loci. All reads mapping to MLST were summed and the percentage mapping to each locus are displayed as a heatmap.

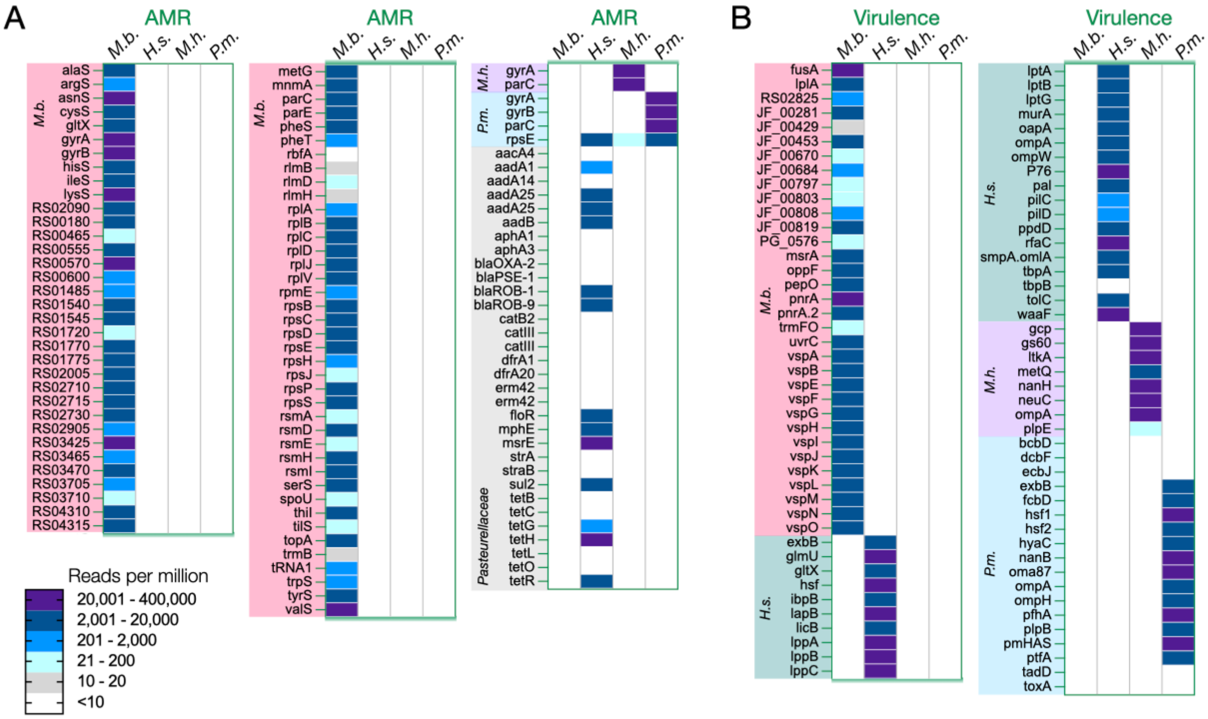

Figure S4: CapSeq detection of ARGs and virulence genes. The percentage of total CapSeq reads from pure cultures mapping to the target sequences in BRD bacterial panel v1. Read mapping to antibiotic resistance loci (**A**) and virulence genes (**B**), colored according to reads per million (RPM) mapping to each locus. Locus names are listed alphabetically in each species. Color coding of locus names is as follows: *M. bovis* (*M.b.*), red fill; *H. somni* (*H.s.*), green fill; *M. haemolytica* (*M.h.*), purple fill; *P. multocida* (*P.m.*), blue fill; and general *Pasteurellaceae*, grey fill.
